# Photoacoustic technologies in nervous system disorders: An emerging strategy for neuromodulation

**DOI:** 10.4103/NRR.NRR-D-24-01191

**Published:** 2025-04-29

**Authors:** Chenyuan Ding, Penghao Liu, Zhuofan Xu, Yuanchen Cheng, Han Yu, Lei Cheng, Zan Chen, Fengzeng Jian, Wanru Duan

**Affiliations:** 1 Department of Neurosurgery, Xuanwu Hospital, Capital Medical University, Beijing, China; 2 Laboratory of Spinal Cord Injury and Functional Reconstruction, China International Neuroscience Institute (CHNA-INI), Beijing, China; 3 Department of Chemistry, Guangdong-Hong Kong-Macao Joint Laboratory of Optoelectronic and Magnetic Functional Materials, Energy Institute and Hong Kong Branch of Chinese National Engineering Research Center for Tissue Restoration & Reconstruction, Hong Kong University of Science and Technology, Clear Water Bay, Kowloon, Hong Kong Special Administrative Region, China; 4 Hong Kong University of Science and Technology-Shenzhen Research Institute, Shenzhen, Guangdong Province, China

**Keywords:** neuromodulation, optogenetics, photoacoustic imaging, photoacoustodynamic therapy, spinal cord injury

## Abstract

Spinal cord injury is a severe neurological disorder; however, current treatment methods often fail to restore nerve function effectively. Spinal cord stimulation via electrical signals is a promising therapeutic modality for spinal cord injury. Based on similar principles, this review aims to explore the potential of optical and acoustic neuromodulation techniques, emphasizing their benefits in the context of spinal cord injury. Photoacoustic imaging, renowned for its noninvasive nature, high-resolution capabilities, and cost-effectiveness, is well recognized for its role in early diagnosis, dynamic monitoring, and surgical guidance in stem cell therapies for spinal cord injury. Moreover, photoacoustodynamic therapy offers multiple pathways for tissue regeneration. Optogenetics and sonogenetics use genetic engineering to achieve precise neuronal activation, while photoacoustoelectric therapy leverages photovoltaic materials for electrical modulation of the nervous system, introducing an innovative paradigm for nerve system disorder management. Collectively, these advancements represent a transformative shift in the diagnosis and treatment of spinal cord injury, with the potential to significantly enhance nerve function remodeling and improve patient outcomes.

## Introduction

Spinal cord injury (SCI) is a severe neurological condition that causes significant motor, sensory, and autonomic dysfunction. A systematic analysis of the global burden of SCI reported an overall incidence rate of 23.77 per million people, with traumatic spinal cord injuries at a rate of 26.48 per million and nontraumatic spinal cord injuries at 17.93 per million. The incidence, prevalence, and years lived with disability increase with age, with males and older adults, particularly those aged > 70 years, experiencing the highest burden. Falls, road injuries, and interpersonal violence remain the leading causes of SCI (Lu et al., 2025). Although the incidence of SCI is decreasing annually with modernization (Lu et al., 2024; Lu et al., 2025), its global burden and complexity remain high. In addition to primary neurological damage, SCI triggers a cascade of multisystem complications, including respiratory failure, cardiovascular dysfunction, urinary tract disturbances, and motor and sensory impairments (Collaborators, 2023). These comorbidities frequently lead to a marked deterioration in the quality of life of affected individuals and contribute to a reduced life expectancy. Consequently, SCI confers substantial health and economic burdens on patients and healthcare systems. Achieving functional restoration after SCI remains a major challenge because of the complexity of the nervous system and the intricate interplay of the biological processes underlying its pathophysiology (Hu et al., 2023).

SCI initiates primary trauma, triggering a cascade of secondary injuries, such as ischemia, oxidative stress, inflammation, and apoptosis, leading to neuronal loss and impaired endogenous regeneration (Anjum et al., 2020; Li et al., 2021b). The therapeutic efficacy of current treatments remains suboptimal because of the intricate healing processes and protective mechanisms of the central nervous system (CNS). Contemporary interventions predominantly fall into two categories: regenerative repair and pro-functional remodeling (Li et al., 2021b). Regenerative repair approaches aim to mimic the natural restorative processes of nervous tissue by replacing lost neuronal function and restoring connectivity, such as removing scar tissue and reconstructing anatomy through stem cell transplantation and biomaterials (Zheng and Tuszynski, 2023). In contrast, pro-functional remodeling enhances the adaptive and compensatory capacities of the residual nervous system, thereby improving overall functionality and patient outcomes, which includes, for instance, spinal cord stimulation (SCS)(McDonald and Sadowsky, 2002; Anjum et al., 2020; Li et al., 2021b). This emerging therapeutic modality, pioneered by Courtine et al., reports its ability to effectively activate spinal cord interneurons, thereby facilitating limb function recovery after SCI (Courtine et al., 2009; Courtine and Sofroniew, 2019; Kathe et al., 2022b; Rowald et al., 2022). However, translational experiments and clinical trials in this field remain limited.

Since the nervous system regulates electrical activity and light and sound can induce electrical conversion, sound and light with certain characteristics may provide broader benefits to the CNS.

The scientific advancements in the 20^th^ century in electricity, magnetism, energy, matter, and vibration have formed the basis for significant developments in light and sound technologies. These modern methods use electrical, optical, and acoustic signals to trigger specific neural activities, offering promising therapeutic potential for various neurological disorders (Li et al., 2024b). Specific acoustic and optical frequencies can elicit distinct neural responses within the nervous system, thereby instigating particular activities. In a compelling interplay of physics and biology, light can be converted into sound through the photoacoustic (PA) effect (Jung et al., 2022). Conversely, light and sound waves can generate electrical energy via photoelectric and sonoelectric effects, respectively (Fu et al., 2019). In addition to their fundamental physical properties, the synergistic application of optical and acoustic technologies extends to integrative approaches that combine these modalities with diverse physical, chemical, and biological elements (Rost et al., 2017; Salehpour et al., 2018; Overchuk et al., 2023). Such interdisciplinary convergence holds the potential to advance novel therapeutic strategies and diagnostic tools, enhancing our ability to study and modulate complex biological systems.

In this review, we explore PA technologies, which integrate the complementary physics of light and sound, exemplified by the PA effect (Yu et al., 2018). This phenomenon, harnessed in our technology, facilitates the generation of acoustic waves in response to light absorption, thereby enabling deep tissue imaging, high spatiotemporal resolution, noninvasiveness, and potential applications in diagnostic techniques and relevant therapeutic neuromodulation (Yu et al., 2018). Individually, photo and acoustic technologies are recognized for their distinct yet synergistic roles in biological systems, integrating photoacoustic imaging (PAI) with emerging therapeutic modalities. Photo technologies leverage light‒biological matter interactions to facilitate processes such as photodynamic therapy (PDT), which is a key approach in medical applications, including cancer treatment (Agostinis et al., 2011). Acoustic technologies, particularly ultrasound, are widely used for real-time, noninvasive imaging and are further explored for their potential in sono-chemodynamic therapy (Lin et al., 2020). Additionally, these technologies can exploit the intrinsic properties of light and sound to generate electrical energy, a concept rooted in photoelectric and sonoelectric effects (Cafarelli et al., 2021; Chen et al., 2022a). These modalities also contribute to immune system enhancement by modulating immune cell activity and strengthening drainage systems to facilitate the clearance of noxious substances, as shown in studies on Alzheimer’s disease and depression spectrum disorders (Hajós et al., 2024; Kim et al., 2024).

**Figure 1:**
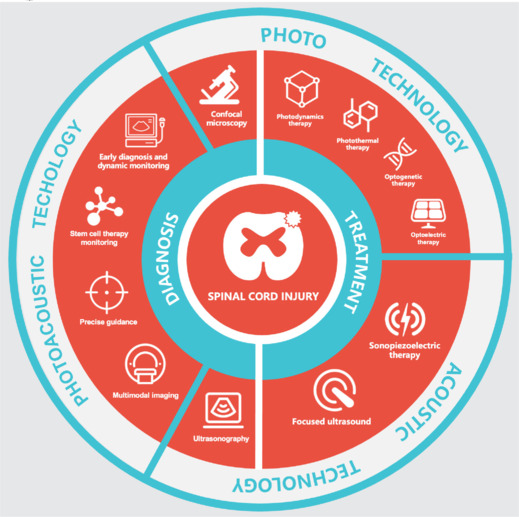
Paradigms of photoacoustic technologies in spinal cord injury. The application of photoacoustic technology to spinal cord injury is first divided into two categories of diagnosis and treatment (white line) and then into three categories (blue line) based on the use of photo and acoustic technology alone or in combination, and specific technologies are filled in the corresponding regions (red area).

Although the spinal cord belongs to the CNS, its mechanisms are different from those of the brain, and research on the spinal cord remains relatively limited (Ceto and Courtine, 2021). Therefore, this review aims to summarize the use of photo and acoustic techniques in the diagnosis and treatment of CNS diseases (**Figure 1**). First, we discuss diagnostic applications, including the use of photo and acoustic techniques—alone or in combination—to visualize the target neural levels. Second, treatment strategies involving different optical techniques and acoustic methods are discussed. Finally, we outline the current limitations and potential resolutions, aiming to identify future research directions and potential clinical applications in SCI.

## Literature Search Strategy

**Figure 2:**
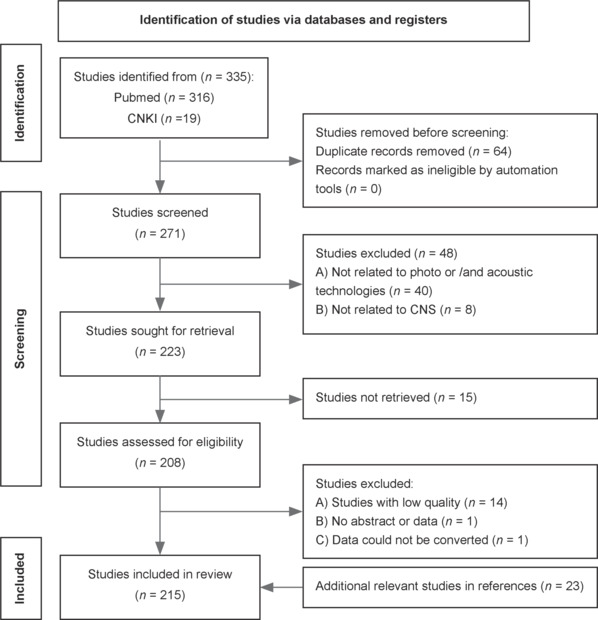
Search procedures and results.

For this narrative review, we conducted a systematic literature search via PubMed (https://www.ncbi.nlm.nih.gov/pubmed) and CNKI (https://www.cnki.net) to identify relevant studies published up to 2024. Our search strategy included keywords such as “spinal cord injury,” “spinal cord,” “photoacoustic imaging,” “photodynamic,” “photothermal,” “photoacoustodynamic,” “photoacoustoelectric,” “optogenetic,” “ultrasound,” “piezoelectric,” and “neuromodulation.” We included only peer-reviewed articles that focused on the CNS, especially the SCI. After removing duplicates from the retrieved studies, we performed a preliminary screening of titles and abstracts, followed by full-text assessments to exclude studies unrelated to CNS and PA technology. In the second round of identification, we applied a snowballing strategy to incorporate additional relevant research PA applications in other systems or treatment effects. This review may be subject to methodology subjectivity and include potential selection bias (**Figure 2**).

## Development of Photoacoustic Technology in Medical Diagnosis

Diagnostic methodologies that rely exclusively on either photo or acoustic methods are widely applied in clinical settings. Optical techniques play a central role in biochemical diagnostics, including but not limited to hematological and urinalysis assays, histopathological microanalysis, and diverse endoscopic imaging modalities (Huang et al., 2019; García-Hernández et al., 2023). Acoustic diagnostics, particularly ultrasound imaging, are fundamental to contemporary medical practice. The utilization of ultrasound for the internal visualization of opaque structures is conventionally dichotomized into four principal categories: A-mode, B-mode, M-mode, and D-mode (Dong et al., 2017). These methods are further enhanced by advanced techniques such as ultrasonic (US) microscopy and acoustic holography, which leverage analogous physical principles governing wave propagation and tissue interaction with biological tissues (Xu et al., 2023).

PAI has emerged as a significant breakthrough at the intersection of PA and biomedicine since its introduction in the mid-1990s, garnering widespread attention across various disciplines and showing immense promise for clinical applications (Grinvald et al., 1986; Zare et al., 2022). The PA effect was initially discovered by Bell in 1880, who reported that modulated light could produce acoustic waves (Erfanzadeh and Zhu, 2019). The development of lasers in the 1960s, characterized by their directional emission, high peak power, and spectral purity, significantly propelled the development of PA techniques. This progress enabled the generation of sound waves in solid materials upon exposure to short-pulsed laser light (Erfanzadeh and Zhu, 2019). These historical developments have led to the sophisticated PAI systems used today, which use the PA effect to achieve high-resolution, deep-tissue imaging (Erfanzadeh and Zhu, 2019; Hosseinaee et al., 2020).

PAI is distinguished by its ability to provide high-resolution imaging, which can be categorized into two main modalities on the basis of imaging principles: photoacoustic microscopy (PAM) and photoacoustic tomography (PAT) (Attia et al., 2019; Seong and Chen, 2020; Chen et al., 2024b). PAM employs a focal-based method to achieve high-resolution imaging, allowing for single-cell visualization by adjusting foci and capturing depth-resolved signals. Conversely, PAT uses unfocused excitation and reconstructs images from diffused energy (Attia et al., 2019; Seong and Chen, 2020; Chen et al., 2024b). It incorporates an array of US transducers to detect ultrasound waves at multiple locations, enabling imaging at high penetration depths of up to 7 cm (Wang and Yao, 2016; Hosseinaee et al., 2020). PAT is versatile, accommodating imaging from microscopic to macroscopic scales (Yao and Wang, 2018). Owing to its exceptional soft tissue contrast and heightened sensitivity to blood volume and oxygen saturation, PAT is frequently utilized for imaging vascular function, diagnosing atherosclerotic plaques, visualizing tumors, and detecting gene expression and protein interactions (Ku et al., 2005; Schoenhagen and Vince, 2014; Aguirre et al., 2018; Merčep et al., 2018; Manohar and Gambhir, 2020; Zheng et al., 2022). Furthermore, advancements in PAI are closely linked to the development of novel exogenous contrast agents, particularly nanomaterials, which enhance PA signals even when endogenous contrast agents, such as blood, are present at low concentrations (Sowers and Emelianov, 2018).

Emerging PA technologies at the intersection of biomedical imaging and optical physics encompass a spectrum of innovative approaches (**Table 1**; Wang et al., 2003, 2011; Xu and Wang, 2005; Wu et al., 2014; Li et al., 2019; Kubelick and Emelianov, 2020c; Hwang et al., 2021; Liu et al., 2021; Ji et al., 2023). These include confocal microscopy, which generates high-contrast images with increased depth resolution for cellular and tissue imaging, and two-photon microscopy, which allows for deep-tissue imaging with reduced photobleaching and phototoxicity, making it ideal for studying neuronal structures and dynamics (Quintá et al., 2015; Liu et al., 2022). Optical coherence tomography serves as a noninvasive imaging modality that provides high-resolution cross-sectional images of biological tissues (Dauerman, 2023). A scanning laser PAM employs a scanning laser to generate PA signals, offering detailed imaging of the microvasculature and other tissue structures (Liu and Yao, 2018). The current literature on scanning laser PAM primarily focuses on technological advancements and experimental validation, with clinical applications still in their early stages. Although scanning laser PAM is more commonly used in cerebrovascular and hemodynamic imaging as well as oncological surgery (Cao et al., 2024; Zafar et al., 2024), studies on its application to the spinal cord remain limited. However, on the basis of this principle, it holds potential for future development in spinal cord imaging.

**Table 1:** Application of photoacoustic technology in the diagnosis of spinal cord injury

Technologies	Principles	Advantages	Disadvantages	References
**Photo technology**				
Confocal microspectral imaging	Apoptosis, bleeding, demyelination, upregulation of chondroitin sulfate proteoglycan and microglia status of injured spinal cord neurons were detected to evaluate the pathological progress	High definition, high resolution, and high magnification, can observe a variety of targets, with three-dimensional imaging ability, which can be quantitatively measured	Small penetration depth and imaging field of view, and equipment costs are high	Li et al., 2019
**Acoustic technology**				
Ultrasound	Visualization of structures within the spinal canal, perfusion imaging, and biomarker imaging can characterize the extent of spinal cord contusion and blood‒spinal barrier breakdown	safe and harmless, convenient for clinical use, and can observe the dynamic changes and predict the prognosis of neurological function in real-time	Strong subjectivity and limited clarity	Hwang et al., 2021
**Photoacoustic technology**				
Early diagnosis	The fluorescence behavior and morphology of neurons were observed by photoacoustic imaging technology, and the early oxygen free radical and glutathione were evaluated in real time to reflect the oxidative stress state of neurons	The real-time diagnosis and monitoring of early spinal cord injury within 24 hours is of great significance to the recovery of therapeutic function	Technical challenges including resolution, depth of penetration, cost and complexity of equipment have not been fully promoted; The clinical issues are the safety of the application, the adaptability of the patient, and the standardization of the procedure	Ji et al., 2023
Dynamic monitor	White matter was observed with specific wavelengths of light and “bond-selective” photoacoustic imaging to evaluate the damage and repair of white matter in the period after spinal cord injury	The penetration depth and imaging field are large, which can realize non-invasive nerve monitoring in vivo during the injury progression		Wu et al., 2014
Stem cell therapy monitor	Real-time guidance of stem cell therapy injection location monitoring using photoacoustic imaging, non-invasive longitudinal monitoring of drug delivery in the spinal cord, and characterization of stem cell function	To minimize the risk of needle shear and accidental damage		Kubelick and Emelianov, 2020c
Precise therapy guiding	Photoacoustic imaging combined with photothermal therapy achieves image-guided targeting and precision guided minimally invasive killing or cutting treatment	It does not produce unnecessary phototoxicity to normal tissues and has advantages in micro-manipulation and precision therapy		Liu et al., 2021
Multimodal imaging	Photoacoustic imaging combined with NMR, tomography, fluorescence imaging, spatial transcriptome and other techniques, in situ microscopy and 3D reconstruction of the whole nervous system including the spinal cord were performed	To achieve a wide field of view, fast and high sensitivity of the whole nervous system fluorescence imaging, imaging samples can maintain the original physical and chemical characteristics and biological activity		Figley et al., 2013

## Potential of Photoacoustic Technology in the Diagnosis and Monitoring of Spinal Cord Injury

Traditional optical methods, such as spectral imaging, are extensively utilized for the pathological examination of SCI, as detailed in subsequent sections on dynamic monitoring. Additionally, perfusion and biomarker imaging are under active investigation to further elucidate post-SCI pathophysiological processes. Ultrasound, another valuable diagnostic tool, facilitates the assessment of spinal cord contusion severity and the extent of blood‒spinal cord barrier disruption in acute SCI. Quantitative ultrasound can be used to monitor the optimal levels of spinal cord blood flow, providing a prognostic indicator for neurological outcomes in porcine SCI models (Fletcher et al., 2020; Hwang et al., 2021). Moreover, PAI integrates the strengths of these imaging techniques, offering enhanced imaging capabilities.

PAI is a noninvasive diagnostic approach that eliminates the ionizing radiation associated with CT scans, thereby minimizing potential harm to the body. Compared with MRI—which, despite its comprehensive imaging capabilities, is time-consuming, causes patient discomfort, requires substantial memory storage, and incurs significant costs (Erfanzadeh and Zhu, 2019)—PAI offers a more practical alternative. Moreover, MRI is limited by its incompatibility with metal implants within the body. In contrast, PAI has widespread clinical utility and convenience over ultrasound. However, unlike conventional ultrasound and noninvasive spinal cord blood flow mapping, which may produce variable image clarity and require subjective interpretation, PAI offers the ability to adjust the laser wavelength. This fine-tuning enables selective tissue visualization and high-resolution, layered three-dimensional reconstruction images that can be crucial for accurate diagnosis (Attia et al., 2019; Hosseinaee et al., 2020). Furthermore, PA technology stands out for its capacity to perform three-dimensional reconstructions, effectively delineating different tissue layers with high definition and resolution, and its ability to capture detailed internal structures at various depths *in vitro*. Its compact, cost-effective design makes it well suited for bedside applications, offering a practical solution for noninvasive diagnostics.

### Early diagnosis

Early diagnosis of SCI within 24 hours, as emphasized in international guidelines, is critical for the prognosis and treatment of patients with SCI. PAI has frequently been utilized in previous studies, capitalizing on the inherent blood flow within vessels to produce high-resolution and high-contrast images (Chen et al., 2024b). Its applications are effective in various medical imaging scenarios, including the depiction of carotid artery disease, visualization of subcutaneous vascular networks in the breast, mapping of the tumor vasculature, imaging of venous structures compromised in systemic sclerosis, and examination of capillary beds (Xie et al., 2020). The ability of this technology to render hemodynamic and microvascular details suggests its potential for detecting microvascular injuries within the spinal cord (Figley et al., 2013; Ji et al., 2023). This hypothesis is supported by the ability of the imaging modality to scrutinize vascular dynamics at the microscopic scale, which is essential for the early identification and intervention of SCI. Current procedures for treating SCI often rely on time-consuming MRI and invasive angiography. PA technologies are suitable for integration into existing diagnostic workflows for SCI. Comparative clinical trials are necessary to verify their accuracy and determine their feasibility for routine clinical use. If proven effective, further evaluation of its safety and potential as a replacement for conventional methods is needed. The potential of the PAI to enhance the early diagnosis and therapeutic management of SCI underscores its value in advancing clinical neurology and biomedical imaging (Aguirre et al., 2018; Jo et al., 2018; Merčep et al., 2018; Oraevsky et al., 2018).

Ji et al. (2023) described the innovative application of PAI technology. They utilized a single-molecule reversible dual PA signal conversion probe, which operates at wavelengths of 680 nm and 750 nm, in conjunction with a selenium-based antioxidant defense nanosystem (Se@BDP-DOH NPs). This system is adept at analyzing neuronal fluorescence behavior, thereby enabling real-time assessment of the oxidative stress state of neurons and glutathione at the SCI site (Ji et al., 2023). Morphological analysis revealed a significant increase in nerve branching and extension with the utilization of Se@BDP-DOH NPs, which promote spinal cord tissue regeneration by increasing neuronal survival, curbing glial scarring, and stimulating the recruitment of endogenous neural stem cells (Ji et al., 2023). These findings suggest that this system has potential for early monitoring and emergency treatment of SCI. However, the cytotoxicity and biocompatibility of Se@BDP-DOH NPs require further verification. The biosafety and detailed mechanisms also need to be carefully investigated before clinical use (Luo et al., 2020; Yuan et al., 2024).

### Dynamic monitoring

Spectral imaging, which is based on optical principles, is widely used in the biological sciences, such as coherent Raman microscopy and confocal microspectral imaging, which are useful tools for CNS diagnosis. The progression of pathological changes after SCI has been meticulously studied using these imaging modalities in conjunction with multivariate analysis (Li et al., 2019). Dynamic monitoring techniques offer critical insights into spinal cord pathologies (Zuser et al., 2010; Heuke and Rigneault, 2023). This approach successfully demonstrated that distinct spectral features and profiles can elucidate key pathological processes that occur post-injury, including neuronal apoptosis, hemorrhage, demyelination, myelin regeneration, and the upregulation of chondroitin sulfate proteoglycans (Li et al., 2019). Furthermore, microglial cell activity, a type of glial cell resident in the CNS, has been quantified in detail by assessing cell density, morphology, process motion, and process length over 14 days following chronic sciatic nerve constriction (Staikopoulos et al., 2021). *In vivo* confocal microscopy enabled real-time visualization of the temporal dynamics of microglial activation in response to peripheral nerve injury.

The limited penetration depth and restricted field of view traditionally associated with the optical imaging techniques described above remain significant challenges in comprehensively assessing extensive tissue damage, particularly in the context of SCI.

However, the advent of bond-selective PAI has overcome this obstacle, offering a novel approach to visualize deeper tissue structures through molecular vibrations with enhanced contrast (Wu et al., 2014; Hui et al., 2016; Pleitez et al., 2020). Wei W et al. pioneered this approach via the use of bond-selective PAI, which is excited at 1730 nm, a wavelength corresponding to the first overtone vibration of the CH2 bond (Wu et al., 2014). Given the abundance of CH2 bonds in the myelin sheath and the high myelin concentration in white matter, this technique provides significantly enhanced PA signal contrast—reportedly twice as strong as that observed in gray matter (Wu et al., 2014). This pronounced contrast provides a distinct advantage for evaluating white matter damage and monitoring reparative processes over time following SCI (Wu et al., 2014). Furthermore, this method allows three-dimensional reconstruction of the entire spinal cord, offering an unprecedented level of detail in visualizing the intricate architecture of the neural tissue (Wu et al., 2014). These findings indicate that PAI can detect morphological changes before and after injury and assess the efficacy of neuroprotective treatments for injured spinal cords (Wu et al., 2014). However, in this report, the lateral and axial resolutions of the PAI are limited by the scanning step size and Nyquist’s law. Potential spinal cord damage from laser-induced heat also needs to be considered (Wu et al., 2014). Future research may focus on the use of a transducer array to increase the imaging speed. Efforts are also underway to develop stabilization equipment to immobilize the spine and mitigate the vibration caused by breath. Furthermore, a higher-power laser system is currently being designed to achieve deeper penetration depth in spinal cord imaging (Wu et al., 2014). The ability to selectively image specific molecular vibrations and convert them into acoustic waves that penetrate deeper into biological tissues represents a paradigm shift in neural imaging. This advancement enables *in vivo*, noninvasive monitoring of nerve fibers during SCI progression and holds significant promise for guiding future interventional therapies (Wu et al., 2014; Hui et al., 2016; Pleitez et al., 2020).

### Treatment monitoring

PAI is an innovative technique well suited for noninvasive guidance in various therapeutic interventions across three principal domains. First, it facilitates precise targeting and resection of diseased tissue through image-guided procedures (Moore and Jokerst, 2019). Second, PA enables continuous treatment monitoring, ensuring real-time observation of therapeutic responses and timely adjustments (Jhunjhunwala et al., 2023). Finally, it enhances the precise delivery of therapeutic agents by leveraging imaging capabilities to ensure that targeted drugs reach the intended sites of action effectively (Zeng et al., 2023). This multifaceted utility underscores the potential of PAI to transform medical diagnostics and therapeutics, offering a comprehensive solution from initial detection to treatment monitoring (Donnelly et al., 2018; Moore and Jokerst, 2019).

PAI enables real-time guidance for precise stem cell injection and noninvasive longitudinal monitoring of drug transport within the spinal cord. Kubelick and Emelianov (2020a, b, c) reported the use of a nanomaterial-based contrast agent for US/PA detection to label spinal cord stem cells. Three-dimensional ultrasound and PAI visualize the distribution of stem cells throughout the spinal cord postinjection, with results comparable to those of MRI in confirming therapeutic effectiveness (Kubelick and Emelianov, 2020c, a, b). This technique holds promise for monitoring cell migration and improving postoperative surveillance of analogous surgical procedures. By enabling accurate, localized stem cell delivery, it minimizes needle-related trauma and enhances therapeutic outcomes (Kubelick et al., 2019). In addition to endogenous contrast agents, James et al. (2021) explored the development and application of exogenous contrast agents in stem cell therapy. Multispectral optoacoustic tomography has emerged as an innovative tool in this domain. The ideal properties of exogenous contrast agents can enable both quantitative and qualitative monitoring of stem cells, further expanding the capabilities of PAI in stem cell research and SCI therapeutic applications (Taruttis and Ntziachristos, 2015; James et al., 2021).

In real-time monitoring of disease treatment—such as in amyotrophic lateral sclerosis, where discrete neuronal populations are affected—ensuring the accurate and precise delivery of stem cells to the ventral horn remains a critical challenge. In this study, the ventral horn was chosen as a target because of its established role in motor neuron disease therapies (James et al., 2021). Jhunjhunwala et al. (2023) introduced a multidisciplinary approach combining nanosensor-augmented stem cell labeling with ultrasound-guided PAI, enabling spatial tracking and functional assessment of transplanted stem cells. This approach represents a significant improvement over current end-point monitoring methods. The use of nanoparticle-based systems may allow for longitudinal tracking of stem cell viability with high spatial and temporal resolution, a capability that other imaging modalities currently lack (Dhada et al., 2019). Experiments targeting each nervous system location should also be conducted accordingly.

In addition, Liu et al. (2021) reported the capacity of PAI when combined with photothermal imaging and therapy, facilitating minimally invasive treatments. This integrated approach allows for targeted positioning and precise guidance within the tumor microenvironment while minimizing phototoxicity to surrounding healthy tissues (Liu et al., 2021). Furthermore, Li et al. (2024a) reported an organic imaging agent activated by white light, which has the highest brightness recorded to date, to improve visual quality. They validated its ability to achieve *in vivo* imaging of liver ischemia‒reperfusion and real-time monitoring during delicate procedures such as kidney transplantation.

These collective endeavors are anticipated to drive progress in therapeutic monitoring and precision medicine, particularly in ultra-sensitive conditions such as those involving spinal cord microvessels and small nerves, where accurate imaging is critical.

### Multimodal medical image fusion of photoacoustic technology

Traditional biomedical imaging techniques, such as positron emission tomography (PET), functional magnetic resonance imaging (fMRI), confocal microscopy, and US imaging, have significantly advanced medical diagnostics (Borsook et al., 2006; Suridjan et al., 2019). Although PET and fMRI can probe deep into biological tissue, their low spatial and temporal resolution limits their ability to distinguish small structures and observe rapid changes. Conversely, optical imaging techniques such as confocal and multiphoton microscopy offer high-resolution images but are constrained by light scattering, limiting their deep penetration in thick tissues (Zhai et al., 2024). The multimodal fusion of PAI with other imaging techniques enhances the detection of neurological diseases but also presents challenges (Li et al., 2023b; Nguyen et al., 2023).

Figley et al. (2013) developed an innovative multimodal imaging model that integrates nuclear magnetic resonance with PAI. This model utilizes an implantable spinal cord window cavity device, facilitating *in situ* microscopic observation of the spinal cord and its vasculature *in vivo*. This approach provides a detailed examination of the microenvironment of the spinal cord, which is crucial for understanding pathophysiological changes post-injury. In parallel, by using an optimally designed image acquisition scheme and a machine-learning algorithm to extract signals from genetically encoded probes through photobleaching-based temporal modulation, the PA can integrate with nuclear magnetic resonance, fluorescence imaging, or transcriptomics. This integration enables high-resolution, three-dimensional isotropic visualization of neural projections in the entire CNS, enhancing diagnostic capabilities (Chen et al., 2024a). Chen et al. (2024a) further developed a novel whole-brain three-dimensional imaging platform known as PATTERN, which is founded on PAT. This platform can achieve a broad field of view with rapid, high-sensitivity fluorescence imaging of the entire nervous system. Additionally, PATTERN preserves the original physicochemical properties and biological activity of the imaging samples, providing an unprecedented level of detail for studying neurological structures and functions.

By leveraging these advanced imaging technologies, researchers can perform a comprehensive, cross-modal analysis of the brain. This approach integrates fMRI, which provides insights into the functional activity of the brain, with high-resolution whole-brain fluorescence imaging, offering cellular and subcellular resolution (Chen et al., 2024a). Additionally, spatial transcriptomics was incorporated, allowing for gene expression mapping across different brain regions. This multimodal integration of these techniques facilitates personalized data coalescence and joint analysis, thereby enhancing our understanding of the complex architecture and function of the brain (Chen et al., 2024a). This integrative strategy aligns well with 3D fluorescence imaging, which is instrumental for studying intricate neural circuits within the brain and spinal cord. This approach is expected to significantly contribute to the future of cross-modal joint analysis in neuroscience, offering novel insights into therapeutic strategies (Chen et al., 2024a).

By fusing multimodal imaging, information can be comprehensively utilized to make up the deficiency of a single mode, which can significantly improve the detection rate and localization accuracy of neurological diseases. Although PAI has advanced considerably, challenges remain in achieving the optimal balance between spatial resolution and imaging depth, as well as in reducing the costs and technical complexity of the technology (Attia et al., 2019). Additional obstacles include data imbalance, difficulties in image fusion, model overfitting, and high costs of equipment and computation (Lin and Wang, 2022; Wang et al., 2023c). Furthermore, establishing standardized protocols for clinical use is essential to ensure patient safety and promote the widespread adoption of this promising imaging modality (Erfanzadeh and Zhu, 2019; Hosseinaee et al., 2020).

Future advancements should introduce innovative fusion methods and technologies, such as adaptive fusion algorithms based on deep learning and real-time multimodal image fusion systems. These developments will enable more comprehensive and accurate descriptions and diagnoses while fostering interdisciplinary research (Zheng et al., 2024).

## Development of Photoacoustic Technology in Medical Treatment

In our therapeutic strategy, we define phototherapy as a treatment that uses light alone and acoustic therapy as one that employs sound exclusively. Based on these definitions, we identify numerous research opportunities. Therefore, we have compiled existing optical and acoustic therapeutic technologies to explore potential research directions for SCI. PA dynamics, a current area of interest, will be discussed, with a focus on PDT and photothermal therapy (PTT). Moreover, the emerging fields of optogenetics and photoelectric therapy are being explored as potential interventions (Ceto and Courtine, 2021; Chen et al., 2022a). Acoustic therapy mainly includes focused ultrasonic therapy and sonopiezoelectric therapy, both of which are under investigation for their potential as novel treatment options (Liba and de la Zerda, 2017; **Table 2**).

**Table 2:** Application of photoacoustic technology in the treatment of spinal cord injury

Technology	Principle	Advantage	Disadvantage	Reference
**High-level laser therapy**				
Laser surgery	It can coagulate, stop bleeding, vaporize or cut tissue by photothermal effect	Combined with a variety of microscopes, stereotactic devices and endoscopes to achieve minimally invasive operation, small size, low power requirements, easy to operate	Thermogenesis is not easily controlled, damages surrounding normal tissue, and vaporized tissue may lead to postoperative edema and sterile inflammation	Devaux and Roux, 1996
**Low-level laser therapy**				
Photobio-modulation therapy	The spontaneous recombination of corticospinal motor circuit after spinal cord injury was improved through mitochondrial mechanism, singlet oxygen mechanism, NO mechanism and macrophage mechanism	Promote nerve cell migration and proliferation without causing irreversible tissue damage, improve dysfunction, and have anti-inflammatory and analgesic effects	At present, there is not enough evidence to show that it is effective in females, and the various influencing factors have not been clearly studied	Ramezani et al., 2020
**Medium-level laser therapy**				
Photo-dynamics therapy	Through photosensitization and oxidation, the photosensitizers can be activated by irradiating the focal sites with specific wavelengths, triggering photochemical reactions to produce ROS, which can effectively reduce the formation of glial scars and promote the re-myelination of injured axons	Precision treatment, large penetration depth, will not cause significant temperature rise in the irradiation area and thermal damage to the tissue, while reducing energy consumption	It is difficult to carry out long-term continuous local light transmission and dose regulation	Li et al., 2011
Photo-thermal therapy	Using photothermal conversion agent nanomaterials, light energy can be converted into heat energy under the irradiation of a specific external light source to achieve functional and organic regulation of nerve cells	It can adjust the redistribution of the conversion agent in the target tissue, with high efficiency, non-invasive, precise regulation of nerve cells and related behaviors in vivo, and has the potential to restore the injured nerve function	The specific mechanism, circulation and metabolic pathway have not been fully defined, and it is difficult to achieve the best design effect and safety guarantee	Zhuang et al., 2023
Opto-genetic therapy	Through gene editing, light sensing genes are transferred into specific cells in the nervous system for ion channel expression, which can selectively excite or inhibit cells under the stimulation of different wavelengths of light	With higher spatial selectivity and specificity, combined with optical implant devices can achieve safe, non-invasive, long-term, wireless optical regulation of the spinal cord, which is of great value for studying the mechanism of neural circuits	May cause neurons to respond beyond the physiological range, resulting in unnatural circuit responses; The expression of light and photosensitive proteins may not be uniform in the target neuronal cell population, resulting in heterogeneity in the amplitude and spatial range of optogenetic manipulation	Ceto and Courtine, 2021
Photo-electric therapy	Using the photoelectric effect, under light irradiation higher than a specific frequency, electrons inside the material absorb energy and escape to form a current, and then use electrical stimulation for positioning, nerve function regulation and precision treatment	Simple, remote, non-invasive, time and space accurate, non-hereditary without genetic engineering, the use of materials generally safe and non-toxic, minimally invasive surgery can be implanted into the corresponding tissue, can be naturally degraded without removal	The body’s response to long-term exposure to medical devices is not fully understood, and the body may also affect the service life of the device, and effectiveness, wearability, and scalability are still being studied	Huang et al., 2023
Focused ultrasound	The use of acoustic lenses or electronic focusing to focus ultrasound waves to a single point to provide concentrated energy can reversibly or permanently modulate nerve function and the blood–spinal barrier through mechanical vibration or thermocoagulation effects	Real-time in vivo regulation with high spatio-temporal resolution, combined with MRI-guided focused ultrasound is a more precise way to treat the blood–spinal barrier to a greater extent and for a longer period of time	Safety and efficacy studies are limited, and further studies are needed to investigate the effects of high-energy FUS on human spinal cord transmission and tissue	Hwang et al., 2021
Sono-piezoelectric therapy	Combined with piezoelectric materials, the nervous system can be electrically stimulated by the effect of sound and pressure. It may regulate neuronal regeneration and compensate electrical communication between neurons by affecting ion transport and neuronal movement	Wireless and non-invasive, low side effects, low lethality to surrounding tissue, low energy attenuation, high tissue penetration	There are few studies on safety and efficacy, and the internal mechanism of ultrasonic excitation of nerve tissue and acoustoelectric materials needs to be further studied	Wang et al., 2023b

### Phototherapy

Phototherapy is a clinical intervention that harnesses light to prevent, treat, and improve various diseases while supporting natural healing mechanisms in the body. It uses white light or artificial light sources, such as infrared, ultraviolet, and visible light, as well as lasers (Ouyang et al., 2022). This modality operates through several mechanisms, including thermal, pressure, photochemical, and photodynamic effects. In laser therapy, clinical applications are categorized based on laser intensity: high-level, low-level, and medium-level laser therapy (Devaux and Roux, 1996; Ramezani et al., 2020). These categories reflect differences in tissue penetration depth and therapeutic applications, offering a tailored approach to light-based treatments for various medical conditions(Diaz et al., 2016).

#### High-level laser therapy

High-intensity laser therapy is utilized primarily for coagulation, vaporization, or cutting in medical procedures, such as femtosecond surgery, prostate treatment, and lithotripsy (Hopstaken et al., 2022). Additionally, it generates pressure and strong electric fields, making it effective in cataract treatment (Lin et al., 2022). In the interaction of lasers with nervous tissue and the CNS, the predominant effect is thermal damage. These procedures include precise hemostasis and dissection of CNS tumors and vascular malformations, as well as applications in stereotactic neurosurgery (Devaux and Roux, 1996).

#### Low-level laser therapy

Photobiomodulation therapy (PBMT), also known as low-level laser therapy, is a noninvasive treatment that is believed to increase oxygen binding to cytochrome c oxidase on the mitochondrial membrane, replacing nitric oxide and potentially increasing ATP production (Nývltová et al., 2022; Zhang et al., 2024). PBMT is also thought to modulate macrophage signals, promoting repair responses and suppressing inflammation. The lasers utilized in this therapy operate in the milliwatt range, with adjustable power and energy densities to prevent irreversible tissue damage (Ramezani et al., 2020). This therapeutic approach stimulates biological processes via lasers to improve physiological, biochemical, immune, and reparative functions. Key applications include promoting wound healing, reducing pain, aiding muscle and nerve tissue regeneration, and providing anti-inflammatory effects (Lutfallah et al., 2023). Kazim et al. (2021) reported the use of PBMT and PDT for SCI treatment. PBMT has the potential to enhance the natural reconnection of corticospinal motor circuits following SCI. This therapy operates through various mechanisms, such as analgesic and anti-inflammatory effects, anti-macrophage activity, and modulation of pain threshold disorders. Consequently, it can effectively reduce the size of the spinal lesion cavity and improve protein and stem cell absorption in the affected area (Kazim et al., 2021). Additionally, PBMT promotes cell migration and proliferation, leading to pain reduction and improved functionality. According to Ramezani et al. (2020), PBMT can effectively improve motor function within 14 days post-SCI, with factors such as light duration, wavelength, energy density, injury severity, treatment regimen duration, and sex influencing outcomes. Furthermore, a study by Sarkaki et al. (2013) revealed that female animals with high progesterone levels do not develop edema. These findings suggest that estrogen and progesterone may protect the nervous system by reducing edema, ischemia, blood‒brain barrier permeability, and neuroinflammation, potentially accelerating post-injury recovery. However, the role of PBMT appeared to be limited in these animals. This highlights the need for further research to explore the underlying mechanisms and effects of PBMT.

#### Medium-level laser therapy

Moderate light intensity has potential for nerve regeneration and repair, particularly in stem cell-related therapy. Li et al. (2014) revealed that 660-nm red light-emitting diode irradiation promotes the migration of bone marrow mesenchymal stem cells, thereby enhancing the efficacy of cell transplantation for treating hypoxic-ischemic brain damage. The therapeutic effect of bone marrow mesenchymal stem cells in treating cerebral ischemia has been confirmed (Li et al., 2016). Potential mechanisms and optimization strategies involve directional migration, differentiation, replacement, neural circuit reconstruction, angiogenesis, neurotrophic factor secretion, apoptosis, and immunomodulation (Li et al., 2016). These findings highlight the significant roles of light stimulation in activating and differentiating stem cells for therapy (Li et al., 2016). Combined stem cell therapy for SCI is also expected to improve the quality of life of individuals and provide substantial social and economic benefits (Ruff et al., 2012).

Advancements in chemistry and materials science have significantly contributed to the progress of PDT and PTT. Innovations in this area include the synthesis of novel dimer photosensitizers, the development of photovoltaic receptors, and the creation of new organic compounds (Wu et al., 2023; Deng et al., 2024). These advancements have led to improvements in conversion efficiency and other key photophysical properties, enhancing the effectiveness of optical diagnostics and therapeutic interventions. Notably, interest in the use of irradiation in the NIR II region (IR-II, 1000–1700 nm) for these therapies is increasing (Zhu et al., 2019). Light within this spectrum offers several advantages, including reducing damage to healthy tissues, increasing penetration depth, and minimizing energy loss (Zhang et al., 2022a). Therefore, treatments utilizing PDT and PTT within this spectral range are gaining recognition for their potential. Furthermore, the integration of these therapeutic modalities with multimodal imaging techniques, such as MRI, is an area of active investigation (Gao et al., 2022a). The synergistic combination of these imaging methods with emerging therapeutic techniques shows promise for diagnostic and therapeutic applications.

#### Photodynamic therapy

PDT is based on photosensitization and oxidation principles. It involves irradiating areas where photosensitizers have preferentially accumulated with specific wavelengths (Li et al., 2020). Upon activation, these photosensitizers initiate photochemical reactions that generate cytotoxic reactive oxygen species (ROS), which selectively destroy diseased tissues (Niu et al., 2021). Modern PDT techniques minimize temperature elevation and prevent thermal damage to the surrounding tissues (Jiang et al., 2019). Consequently, PDT was employed to treat various conditions, including neoplastic tumors, precancerous lesions, proliferative skin disorders, vascular malformations, and other pathologies (Li et al., 2020), making it a versatile and minimally invasive therapeutic option.

Wright et al. (2009) revealed the neuroprotective potential of PDT by selectively targeting tissues while sparing neurons. Their study revealed that human adenocarcinoma MCF-7 cells and satellite glial cells were significantly more sensitive to PDT than were neurons, resulting in the effective elimination of tumor and satellite glial cells with preserved neuronal viability.

In SCI management, PDT has also been investigated. Li et al. (2011) proposed that since PDT reduces glial scarring, only cells close to the ROS-producing area are destroyed. Therefore, they hypothesized that it might be possible to control the range of PDT to inhibit SCI glial scars without destroying normal tissue. This was supported in 2016 when the use of polyethylene glycol combined with the photosensitizer UCNPs-PEGM 540, in conjunction with PDT, confirmed this hypothesis (Liu et al., 2016). *In vitro* experiments indicated that exposure to 980 nm near-infrared (NIR) light enabled upconversion nanoparticles to effectively ablate astrocytes and foster myelin restoration (Liu et al., 2016). This study revealed that reducing glial scar formation and promoting myelin regrowth in damaged axons could enhance functional recovery in the hind limbs of rats. The regenerating axons in the corticospinal tract bypass the injured area, reaching the caudal pulp and ameliorating the surrounding microenvironment (Liu et al., 2016).

PDT requires stable fixation of the optical device to the internal tissue surfaces for continuous, localized, low-dose, long-term light delivery. Yamagishi et al. (2019) developed a wirelessly powered, implantable device with a near-field communication-based light-emitting diode chip and a bioadhesive, stretchable polydopamine-modified poly(dimethyl-siloxane) nanosheet, which was securely anchored to the inner surface of the targeted tissue. This subdermal device provided significant antitumor irradiation over 10 days at an intensity approximately 1000 times lower than that of conventional PDT. This innovation holds the potential for treating deep lesions that are challenging to detect and access (Yamagishi et al., 2019).

#### Photothermal therapy

PTT is a noninvasive cancer treatment that utilizes the photothermal effect, where laser energy is converted into heat by photothermal agents to selectively destroy tumor cells while minimizing damage to surrounding healthy tissues. PTT employs nanomaterials capable of efficiently converting absorbed light, particularly from the NIR spectrum, into heat, exploiting the thermal tolerance difference between cancerous and normal cells (Chen et al., 2020, 2021b). Although PTT is distinct from PDT, PTT can be synergistically combined with PDT to improve therapeutic effects (Nomura et al., 2020).

For instance, breast cancer cell proliferation was effectively inhibited, and tumor recurrence post-PTT was significantly reduced (Ning et al., 2022). However, a key challenge remains: accessing the targeted tissue while minimizing collateral damage to surrounding healthy tissues. To address this, Mooney et al. (2014) proposed a novel approach involving the neural stem cell–mediated delivery of gold nanorods for PTT. This method enhances the intratumoral delivery of agents for treating triple-negative breast cancer, increasing the effective concentration at the tumor site without affecting adjacent healthy tissue and thereby improving therapeutic efficiency.

In the context of neurological tumors, PTT shows enhanced spatiotemporal resolution under NIR-II irradiation, enabling *in vivo* detection of gliomas through fluorescence imaging (Li et al., 2023a). This advancement has facilitated the development of a drug delivery nano-platform for real-time monitoring of the delivery process, which is crucial for targeted drug therapy in gliomas while also improving blood‒brain barrier permeability (Qian et al., 2018; Li et al., 2022a). Furthermore, photothermal-mediated chemotherapy and chemodynamic therapy have been proven to effectively inhibit glioma proliferation (Li et al., 2021a). Similar therapeutic effects have been observed in malignant peripheral nerve sheath tumors (Sweeney et al., 2016; Gu et al., 2022).

In neurodegenerative diseases, PTT shows significant potential in addressing diverse neurological conditions. Nanosystems with strong photothermal properties dissolve fiber precipitates under NIR light, significantly improving blood–brain barrier permeability. This facilitates drug delivery across the blood-brain barrier and overcomes the limitations of traditional anti-AD drugs. Additionally, the interaction between photosensitizers or photothermal transducers and light can trigger photochemical reactions, producing reactive oxygen species or thermal effects that regulate amyloid-beta (amyloid-beta peptide polymerization into fibrils/plaques), significantly reducing amyloid-beta deposition and neuroinflammation and improving learning and memory deficits. PTT offers advantages such as operational flexibility, noninvasiveness, and high spatial and temporal resolution (Sweeney et al., 2016; Zeng et al., 2021; Ye et al., 2023). Gao et al. (2022b) reported that, guided by the photothermal effect, MgOp@PPLP nanoparticles can penetrate the blood‒brain barrier and be taken up by neuronal cells for gene therapy and antioxidant therapy. In both *in vivo* and *in vitro* models of Parkinson’s disease (PD), MgOp@PPLP has strong neuroprotective effects(Gao et al., 2022b). Similarly, (Liang et al., 2024) revealed that PTT, coupled with responsive levodopa release and reduced ROS levels in the CNS, enhances drug delivery across the blood‒brain barrier. In combination with noninvasive NIR or NIR-II radiation, nanoplatforms with good biocompatibility are emerging as ideal materials for combating PD. Nedosekin et al. (2021) also highlighted their theranostic platform integrating PTT, which directly disrupts misfolded protein accumulation, maintains motility, and extends the lifespan of treated nematodes. Coupled with advanced imaging to monitor the photothermal ablation of aggregates, this platform initiates systemic recovery, validating the concept of aggregate-disruption treatments for Huntington’s disease in humans.

In neuromodulation, Zhuang et al. (2023) developed an NIR PTT technique using the nematode Caenorhabditis elegans as a model. They reveal that cellular excitability and animal behavior are regulated by the photothermal agent indocyanine green through the thermosensitive transient receptor potential vanilloid 1 channel. Upon NIR stimulation in the presence of indocyanine green, exogenous expression of transient receptor potential vanilloid 1 in sensory neurons induces a Ca^2+^ influx, heightening neural excitability and reversing behaviors. Furthermore, NIR irradiation activates GABAergic D-class motor neurons, dampening pulsating movements (Zhuang et al., 2023). Jung et al. (2021) developed an innovative artificial extracellular matrix by integrating gold nanocages and showed its ability to promote neuronal differentiation when cocultured with rat neural stem cells under PTT irradiation.

In the peripheral nervous system, PTT also effectively activates neurons. Jiang et al. (2023) developed a novel PTT-controlled release immunomodulatory hydrogel nanoplatform that integrates polydopamine-reduced graphene oxide with thermal-sensitive properties. This platform has been successfully applied to treat peripheral nerve damage in infectious diabetic ulcers, showcasing its potential in nerve repair strategies (Jiang et al., 2023). Recruited Trem2^+^ macrophages regulate collagen remodeling, repair skin adnexal structures, alter scar formation, promote angiogenesis, and support the regeneration of peripheral neural networks (Jiang et al., 2023). Additionally, light and heat effectively disrupt biofilms, kill various microorganisms, and eliminate bacteria without inducing drug resistance (Chen et al., 2020; Huo et al., 2021).

Currently, these techniques are mainly restricted to basic animal model studies, with ethical concerns surrounding their use in clinical trials. Therefore, demonstrating efficacy in large animals and humans remains the primary focus of future research.

#### Optogenetics therapy

Optogenetics is an interdisciplinary bioengineering technique that combines optics, genetic manipulation, and electrophysiology to precisely control and monitor cellular activities (Rost et al., 2017). Channelrhodopsin-2 (ChR2), a non-selective cationic channel protein with a 7-transmembrane structure, has been a key tool in optogenetic research since its discovery in Chlamydomonas reinhardtii in 2003 (Volkov et al., 2017). This light-sensitive protein undergoes a photocycle upon photon absorption, transitioning from a dark state to an active state, allowing ion flow across the cell membrane (Volkov et al., 2017). Boyden et al. (2025) first expressed the light-sensitive channel protein on mammalian neurons, which responded to light with electrical activity, and the firing of neurons depends on the time and intensity of light. It marked a pioneering step in optogenetics. Since 2010, the advancements in optogenetics have been significantly propelled by the development of high-quality lasers, enabling more precise control over neuronal stimulation and silencing (Ceto and Courtine, 2021; Karatum et al., 2023).

The core principle of optogenetics involves the initial use of genetic manipulation to introduce light-sensitive genes—such as ChR2, enhanced bacterial rhodopsin, halorhodopsin 3.0, archaerhodopsin, or OptoXR—into targeted cell types in the nervous system, thereby expressing specific ion channels (Rost et al., 2017). These photosensitive ion channels, activated by different light wavelengths, selectively conduct cations or anions with high spatial selectivity, altering the transmembrane potential and facilitating the selective excitation or inhibition of neurons (Jiang-Xie et al., 2024).

Optogenetics has been widely applied across various research disciplines, including neural circuitry analysis, learning and memory studies, PD model construction, and multi-species models for investigating depression and anxiety disorders (Tye et al., 2013; Rajasethupathy et al., 2016; Kim et al., 2023a). Recent advances have expanded to the transfection of photoreceptor channel protein genes into neurons within the corticospinal tract following trauma (Montgomery et al., 2015; Samineni et al., 2017; Spencer et al., 2018). Upon photoactivation, these genes trigger responses similar to those induced by dorsal root stimulation, enhancing our understanding of mechanisms in the afferent nervous system, such as sensory perception and visceral pain experience (Montgomery et al., 2015; Samineni et al., 2017; Spencer et al., 2018).

Most studies focus on motor function recovery. For instance, ChR2 utilization has substantiated that light stimulation of the diaphragm motor nucleus below a cervical SCI can reinstate respiratory movements (Alilain et al., 2008). In mice with codeletion of the PTEN and SOCS3 genes, light irradiation of corticospinal tract fibers in the cervical spinal cord has been shown to induce bilateral forelimb movements (Jin et al., 2015). Furthermore, light stimulation of the ventral projection in mice with SCI, combined with anti-inflammatory interleukin-10 therapy, induces a strong forelimb muscle response (Chen et al., 2021a).

Research into visceral reproductive functions has been more limited. Qi et al. (2024) employed the implantation of light-sensitive channel genes to investigate how genital signals trigger circuits between the spinal cord and brain, leading to sexual arousal. The study reveals that shining light on neurons expressing these light-sensitive channels could replicate mechanically induced sexual reflexes in mice (Qi et al., 2024). Additionally, Mickle et al. (2019) described a miniaturized bio-optoelectronic implant that wirelessly regulates the peripheral nervous system in a closed-loop manner to achieve stable, targeted sacral nerve stimulation. This technology is designed for treating conditions such as overactive bladder, urinary incontinence, and interstitial cystitis.

Electrode designs for optogenetics are similar to those used in SCS and can be categorized into three types. The first type involves an external light source connected through optical fiber transmission. This method requires a stable external light source but limits patient mobility and comfort due to the physical connection (Huang et al., 2020). The second type is affixed to the skin, and it typically receives power through wired connections. Although this design allows for some wireless operation, it remains tethered to an external power source (Kim et al., 2021). The third type is fully internal, utilizing wireless transmission to power an internal light source. Technologies such as near-field energy transmission, far-field radiation energy transmission, US power supply, and kinetic energy harvesting are crucial for the convenience of wireless devices, as they eliminate the need for frequent battery replacements and reduce the invasiveness of implantation procedures (Mickle et al., 2019; Metuh et al., 2024). These different implants provide a strong foundation for further exploration.

**Figure 3:**
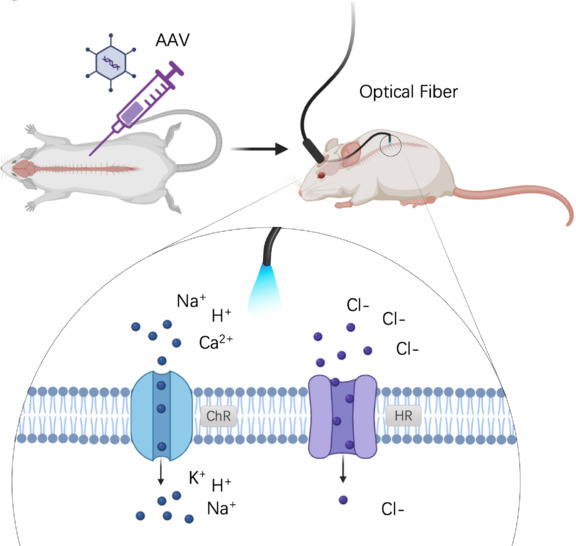
Schematic diagram of optogenetic mechanisms and experimental processes. Created with BioRender.com.

In the field of SCI, Kathe et al. (2022a) successfully positioned a micro-LED above the dorsal horn to facilitate precise light stimulation of afferent fibers and spinal interneurons. Stimulating the caudal lumbar region with light has elicited strong muscular responses in the tibialis anterior muscle, leading to significant dorsiflexion of the foot (Kathe et al., 2022a). Additionally, this device has provided insights into the roles of various neuronal subtypes, sensory pathways, and spinal projections in motor control in both healthy and mice with SCI (Kathe et al., 2022a). In another study, Mondello et al. (2021) introduced a long-term implantable micro light emitting iode (LED) device for spinal cord photogenetic stimulation in awake, freely moving rats. This device successfully induced sustained vigorous movement for at least 6 weeks without causing physical or thermal damage to the underlying spinal cord. The application and principle of optogenetic implantation devices in rats are shown in **Figure 3**.

The application of optogenetics and PA techniques for precise neural activity control and neural circuit studies represents a promising direction for further exploration. Identifying and manipulating different neuron classes in behaving animals is crucial for understanding their roles in circuit dynamics and behavior (Roux et al., 2014). Beyond optogenetics, these technologies offer therapeutic potential in PDT and PTT, along with deep-tissue biosensing.

Shcherbakova et al. (2018) explored the use of monomeric NIR fluorescent proteins and NIR biosensors for multiplexed imaging alongside commonly used green fluorescent protein-based probes and blue light-activatable optogenetic tools. These NIR probes enable visualization of functional activities across molecular to organismal scales. When combined with advanced imaging techniques, such as two-photon microscopy with adaptive optics (PAT) and its recent modification, reversibly switchable photoacoustic computed tomography (PACT), NIR probes enable subcellular resolution at millimeter-scale depths and serve as powerful biosensors for deep-tissue imaging (Shcherbakova et al., 2018). Furthermore, advancements in optogenetic technology using photosensitive proteins offer insights into how dynamic information is transmitted and interpreted within complex networks of intercellular and intermolecular interactions. The combination of microscopy and optogenetics provides valuable insights into the mechanisms underlying dynamic information processing at the single-cell level (Isomura and Kageyama, 2017).

The emergence of cardiac optogenetics and its potential clinical applications is a key area of research. This field combines optogenetic manipulation with optical voltage measurement to achieve real-time control of cardiac electrophysiology, particularly in the treatment of specific arrhythmias. Additionally, the development of high-throughput electrophysiological assays, alongside emerging techniques such as PAI and stress sensors, highlights promising strategies for future research advancements (Müllenbroich et al., 2021). Insights gained from cardiac optogenetics may inform research on the nervous system, as both share a foundation in electrophysiology.

#### Photoelectrotherapy

The production of photoelectricity primarily depends on the photoelectric effect. When certain materials are exposed to electromagnetic waves—such as light—with a frequency higher than a specific threshold, their electrons absorb energy and transition out of their original energy state, leading to an electric current known as photoelectricity (Wei et al., 2024). The electrophysiological effects of direct current can enhance tissue excitability, improve membrane permeability, regulate the acidic microenvironment, and promote tissue hydration (Qi et al., 2022). Photoelectric conversion is typically achieved using light-responsive electrodes, generating direct current or pulsed signals at low, medium, and high frequencies (Lu et al., 2016; Chen et al., 2022a; Meng et al., 2022).

Although the photoelectric effects have been utilized in peripheral nerve treatment, their application in the CNS remains largely underexplored. The fundamental principle involves using functionalized nanoparticles, monocrystalline silicon, and other photovoltaic materials to convert light into electrical current, facilitating nerve stimulation through charge redistribution (Huang et al., 2023; Zhang et al., 2023a). Beyond the lasers, these systems can integrate other external stimuli, such as acoustic, electrical, or magnetic signals, facilitating signal interconversion or chemical reactions(Yang et al., 2024). This process is essential for regulating stimulation-sensitive ion channels and ion migration, both of which are crucial to nervous system function (Yang et al., 2024). Sun et al. (2024) developed a compact photoelectronic device based on a thin-film silicon (Si) diode, recognized for its complete biodegradability and flexibility. The device was further optimized with an interface modification layer composed of degradable molybdenum (Mo) and extended electrodes, enabling tripolar stimulation to improve charge injection efficiency and overall stimulation efficacy. The neural regulation was effectively achieved by irradiating deep red light (635 nm) onto the sciatic nerves of Sprague-Dawley (SD) rats and the facial nerves of New Zealand rabbits, successfully restoring the function of damaged nerves through transdermal photostimulation (Sun et al., 2024). Li et al. (2024c) developed a wireless photoelectric device fabricated from a monocrystalline silicon film. This minimally invasive implant harnesses light to regulate cardiac pacing with high precision. The silicon film is designed to naturally degrade into non-toxic silicate within the body. These innovative designs offer promising strategies for modulating neural activity and hold significant potential for future applications in SCI treatment (Li et al., 2024c; Sun et al., 2024).

### Acoustic-based therapy

The use of ultrasound for treating SCI has been explored less extensively than optical therapies. Therapeutic ultrasound influences the nervous system by reducing nerve excitability and slowing conduction velocity at low intensities, resulting in a significant analgesic effect in peripheral nerve conditions such as neuritis and neuralgia (Narouze, 2016). Furthermore, ultrasound can enhance drug transport by increasing biofilm permeability through mechanisms such as diffusion, thixotropy, cavitation, polymerization, and depolymerization (Zhang et al., 2022b). Additionally, ultrasound treatment elevates intracellular Ca^2+^ levels, activates fibroblasts, enhances protein synthesis, increases vascular permeability, facilitates blood vessel formation, strengthens collagen tension, boosts enzyme activity, and induces pH changes that mitigate inflammation (Beisteiner et al., 2023; Yamaguchi et al., 2023; Seasons et al., 2024). These effects collectively contribute to nerve tissue repair and regeneration (Fletcher et al., 2020; Hwang et al., 2021). For example, Zhang et al. (2023a) reported that US-induced Piezo1 downregulation is the key mechanism through which combined therapy promotes neural stem cell differentiation into neurons and exerts anti-inflammatory effects. Additionally, the Piezo1/NF-κB signaling pathways were identified as critical to these processes. Piezo1, a piezo-type mechanosensitive ion channel component 1, is crucial in low-frequency sonophoresis-induced mechanotransduction pathways, which activate downstream cellular signaling processes (Jiang et al., 2024). This study demonstrated that combining ultrasound with functional nanoparticles represents a promising strategy for injured spinal cord repair.

Recent evidence suggests that ultrasonic waves can activate ion channels, thereby altering the electrophysiological properties of targeted neurons (Jiang et al., 2024). Fan et al. (2024) characterized the excitatory effects of low-intensity pulsed ultrasound on spontaneous activity and intracellular Ca^2+^ homeostasis in cultured hippocampal neurons, providing insights into the mechanisms underlying low-intensity pulsed ultrasound-induced neuroregulation. This review will specifically focus on the investigation of focused ultrasound (FUS) and sonopiezoelectric therapy.

#### Focused ultrasound

FUS functions by directing ultrasonic waves to a single focal point through pre-calculated interference (Rabut et al., 2020). In the CNS, FUS plays several roles, including modulating vascular permeability, regulating neuronal activity, providing neuroprotection, and facilitating localized therapies, making it a promising approach for SCI treatment (Hwang et al., 2021). FUS is classified into high-intensity focused ultrasound, which induces irreversible tissue ablation, and low-intensity focused ultrasound, which produces reversible physiological effects (Darrow, 2019). Low-intensity focused ultrasound has gained recognition as a promising technique for reversible neuromodulation and transient disruption of the blood–brain and blood–spinal barriers, offering potential therapeutic applications for various neurological conditions (Zhong et al., 2023).

MRI-guided FUS is a precise and noninvasive approach for promoting SCI recovery. This technique leverages microbubble cavitation to target the injured spinal cord, whereas MRI enables real-time visualization and measurement of blood‒spinal cord barrier permeability (Lamsam et al., 2018; Cross et al., 2021). Rincon-Torroella et al. (2022) suggested that at lower intensity and pulse frequency, MRgFUS might briefly breach the blood‒brain barrier. Thus, while facilitating access to standard or novel therapies within the tumor or parenchyma, MRgFUS-mediated disruption of the blood‒brain barrier opens up the possibility of enhanced detection of biomarkers of brain tumor origin. This technology, previously used to treat brain tumors, epilepsy, and PD, has potential for ablating diseased tissue and facilitating the delivery of targeted drugs, including chemotherapy and immunotherapy, across both the blood‒brain and blood‒spinal cord barriers (Weber-Adrian et al., 2015; Cross et al., 2021; Rincon-Torroella et al., 2022).

#### Sonogenetics

Sonogenetics, an emerging medical technology, employs acoustic waves to regulate cellular activity through genetically encoded sonosensitive mediators. This method enables remote and noninvasive modulation of specific molecular processes and biomolecular functions (Wang et al., 2023a). Sonogenetics offers the potential for precise spatiotemporal control in gene- and cell-based therapies owing to its intrinsic benefits, including noninvasiveness, high safety, and deep tissue penetration abilities (Hahmann et al., 2024). Additionally, sonogenetics has various applications, including tumor immunotherapy, the mitigation of Parkinsonian symptoms, the modulation of neural reward pathways, vision restoration, and stem cell therapies (Tang et al., 2024; Wu et al., 2024). The method is gaining prominence along with optogenetics, electrogenetics, and magnetogenetics, highlighting the significance of noninvasive and precisive cellular manipulation techniques (Wang et al., 2023a).

#### Sonopieelectric therapy

The piezoelectric effect enables the conversion of mechanical or acoustic energy into electrical energy, facilitating remote electrical stimulation through ultrasonic power (Deng et al., 2022). This phenomenon occurs when a material deforms under mechanical stress, generating electronic polarization—a process known as the direct piezoelectric effect. Conversely, when an electric field is applied along the polarization axis, it induces material deformation, a mechanism known as the converse piezoelectric effect (Stetsovych et al., 2018). Sodium niobate (NaNbO3) is a material that exhibits both sonopiezoelectric and photoelectric semiconductor properties (Wang et al., 2021; Chen et al., 2022b). These characteristics enable passive electrical stimulation, offering a mechanism for modulating the electrical properties of materials in response to applied mechanical stress or electric fields.

Sonopiezoelectric technology (due to its ability to modify surface charge) can attract significant numbers of drug-loaded microcapsules (Kim et al., 2023b). This technology facilitates precise, controlled drug delivery, enabling the transport of antibiotics, anti-inflammatory drugs, transdermal glucose administration, and enhancing tumor treatment efficacy by combining ultrasound with chemotherapy (Li et al., 2022c; Xu et al., 2024). Additionally, acoustic piezoelectric materials can generate ROS within cells through their internal electric field, contributing to apoptosis in tumor cells. When combined with antibodies, these materials facilitate targeted tumor therapy (Deng et al., 2024). The integration of the sonopiezoelectric material barium titanate with anti-HER2 antibodies enables the selective inhibition of human epidermal growth factor receptor 2 (HER2)-mediated cell proliferation, demonstrating potential applications in the treatment of SCI (Pucci et al., 2022).

Sonopiezoelectricity is commonly used in the functional regeneration of the nervous system. This technology promotes neuronal regeneration by modulating ion transport, guiding neuronal migration, and facilitating electrical communication between neurons (Wang et al., 2023b). A key advantage of sonopiezoelectricity is its wireless and noninvasive nature, enabling precise neural modulation. For example, stimulating SH-SY5Y neuron-like cells can be achieved by irradiating barium titanate, which induces Ca^2+^ influx through voltage-gated channels. This process promotes neurite outgrowth and neuronal differentiation (Pucci et al., 2022). The underlying mechanism involves stimulating synaptic regeneration and nerve cell elongation by regulating Ca^2+^ influx. Additionally, it promotes PC12 cell differentiation through cyclic adenosine monophosphate (cAMP)/protein kinase A (PKA)-dependent pathways (Hoop et al., 2017). Sonopiezoelectricity regulates ROS production, modulates the immune response during nerve repair, and maintains energy balance (Du et al., 2024). Furthermore, it induces the differentiation of bone marrow mesenchymal stem cells into neurons, demonstrating its potential for autologous stem cell therapy in nerve repair and regeneration (Zhang et al., 2023b). This technology holds promise for the treatment of PD and Alzheimer’s disease.

Ultrasound offers unique advantages over optical technologies in sonodynamic and sonothermal therapies. PA, which combines the benefits of both optical and acoustic modalities, is expected to serve as a valuable complement to existing clinical practices. Integrating PA with various medical techniques is projected to enhance diagnostic and therapeutic capabilities while creating new opportunities for addressing the complex demands of biomedical applications (Li et al., 2022b). In summary, the integration of PA into clinical medicine represents a significant advancement in diagnostic and therapeutic interventions. As research continues to reveal the full potential of this technology, PA could become an essential component of the biomedical toolkit, enhancing patient care and clinical outcomes.

## Future Expectations

Translational medicine has emerged as a key concept in the 21^st^ century, facilitating a more direct connection between basic research and clinical practice. PAI technology has rapidly advanced as a biomedical multi-wave imaging method (Valluru and Willmann, 2016). In recent years, its ability to detect malignant tissue has garnered significant attention, particularly for its advantages in tumor surgery (Zare et al., 2022). PAI has shown promise in several biomedical applications, monitoring angiogenesis, evaluating blood oxygen saturation, conducting CNS functional imaging, assessing cortical blood volume, detecting skin and conjunctival melanoma depth, determining methemoglobin levels, tumor hypoxia, and lymph node metastasis (Wang et al., 2003; Shao et al., 2013; Yao and Wang, 2014; Rich and Seshadri, 2015; Khattak et al., 2019). Additionally, PAI enables real-time intraoperative and multimodal imaging (Zhou et al., 2009). Several generations of PAI systems have been developed, including bedside imaging machines designed for clinical use. FDA-approved PAI is advancing into clinical settings for oncology applications, including head and neck cancers, thyroid nodules, breast carcinomas, gynecological cancers, melanoma, rectal lesions, and prostate carcinoma (Aguirre et al., 2011; Cârțână et al., 2016; Lydiatt et al., 2017; Yamaga et al., 2018; Xia et al., 2019; Zare et al., 2022).

This review highlights the advantages of super-resolution in PAI, the integration of fiber optic technology in PAI systems, and the development of multimodal systems combining PAI with other photonic or acoustic therapies. However, challenges such as high equipment costs, operational complexity, and the need for clinical standardization remain unresolved.

The device is compact and bedside-accessible, offering a cost-effective solution; however, its development may involve additional expenses. Technical limitations include restricted penetration depth and reliance on specific wavelengths. Furthermore, challenges remain in translating preclinical findings into human clinical trials.

That technique is being refined for brain and visual detection trails to enhance spatial resolution, reduce motion artifacts, and mitigate skull or vertebrae-induced attenuation (Zare et al., 2022). Consequently, the probe must be positioned close to the patient, which can compromise patient evaluation by introducing safety concerns such as risks associated with laser-induced acoustic waves, heat generation, and other potential hazards. A common limitation of all brain imaging systems is the need to rotate the detector array around the patient to capture multiple projections for tomographic reconstruction, a process that is time-consuming and susceptible to degeneration in image quality due to patient movement (Zare et al., 2022). Therefore, optimizing specific geometries is essential to obtaining a comprehensive set of projections. Potential safety concerns related to patient adaptability and long-term adverse effects remain unclear. Establishing standardized operating procedures and implementing safety regulations in a timely manner is necessary.

The development and targeted application of novel endogenous and exogenous contrast agents using advanced engineering and emerging technologies remain challenging, limiting the adoption of PAI for CNS applications. However, exogenous contrast agents have proven beneficial in tumor diagnosis and treatment, contributing to reduced mortality. Future studies should focus on developing more efficient stimuli-responsive agents capable of tracking shifts in absorption peaks based on changes in the biological microenvironment while evaluating their toxicity, biocompatibility, biodegradability, and photostability. Overcoming these challenges is essential for advancing the clinical application of therapeutic diagnostics and strategies in the CNS. Further engineering and translational studies are required to advance the field. Additionally, deep-learning strategies for optimizing image reconstruction may hold promise for future applications. The precise implementation of optogenetics and the continued investigation of its underlying mechanisms are closely associated with advancements in genetics and molecular biology.

Finally, interdisciplinary research on PA technology in neuroscience integrates materials science, genetics, molecular biology, and computer science, among other fields. Expanding collaborative efforts in these fields could broaden its applications and enhance its potential. This review highlights the novelty of combining PAI as a diagnostic strategy with emerging therapeutic modalities while outlining potential future research directions. Additionally, the application of this technology can extend to biological, environmental, chemical, and material sciences, enhancing its scalability and influence.

## Limitations

This review outlines the fundamental physics underlying PA techniques for measuring and manipulating different neural pathways. However, this review has some limitations. It primarily focuses on basic experimental design and engineering principles without providing significant practical applications. Additionally, it lacks a comprehensive discussion of data processing methods. This review may not provide a fully comprehensive assessment of research advancements in PA technology for neurological diagnosis and treatment. Furthermore, the potential clinical translation of these techniques requires further discussion.

## Conclusion

This narrative review highlights the multifaceted applications of photoacoustic technologies in diagnosing, monitoring, and treating SCI. PAI has emerged as a transformative modality, offering noninvasive, high-resolution, and deep-tissue imaging capabilities that significantly enhance early diagnosis and ongoing monitoring of SCI. By integrating the strengths of optical and acoustic techniques, PAI provides detailed insights into the pathological progression of SCI, facilitating real-time assessment and precise guidance of therapeutic interventions.

**Figure 4:**
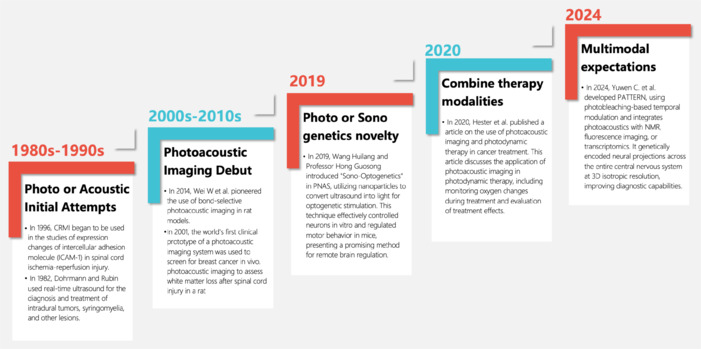
Chronology of important milestones in photoacoustic technologies. The data are sourced from Rubin et al., 1988; Wu et al., 2014, 2019; Li et al., 2019; Chen et al., 2024a; Menozzi and Yao, 2024; Patil-Takbhate et al., 2024; Wang et al., 2024.

Beyond imaging, photoacoustic technologies demonstrate substantial therapeutic potential. Emerging innovations—such as photoacoustic dynamic therapy, opto- or sono-genetics, and photo- or sono-electric therapy—are paving new avenues for neural repair and functional remodeling. By harnessing the unique properties of light and sound, these technologies can modulate neural activity, attenuate inflammation, stimulate axonal regeneration, and ultimately enhance overall recovery outcomes. Integrating these modalities with advances in genetic engineering and cutting-edge materials further broadens their application potential, offering new avenues for personalized medicine and targeted therapeutic strategies. The important milestones in photoacoustic technologies are shown in **Figure 4**.

Looking to the future, several promising research directions emerge. First, developing novel contrast agents and imaging systems to enhance the resolution and depth of PAI is crucial for its clinical translation. Second, optimizing the delivery and activation of therapeutic agents—such as photosensitizers and nanoparticles—will be essential for maximizing the benefits of PDT and PTT. Third, integrating photoacoustic technologies with emerging fields such as artificial intelligence and nanotechnology could lead to more sophisticated diagnostic tools and personalized treatment protocols. Finally, conducting large-scale clinical trials is necessary to validate the safety and efficacy of these technologies and to facilitate their integration into mainstream clinical practice.

In conclusion, photoacoustic technologies represent a significant advancement in SCI research and treatment. Their unique ability to merge high-resolution imaging with targeted therapeutic interventions positions them as a cornerstone for future innovations in neurotrauma management. Continued research and development in this area hold the potential to revolutionize SCI care, offering new hope for patients and healthcare providers alike.

## Data Availability

*Not applicable*.
